# Cell cycle arrest biomarkers for early diagnosis of acute kidney injury after liver transplantation: A prospective cohort study

**DOI:** 10.1097/EJA.0000000000002123

**Published:** 2025-01-22

**Authors:** Benjamin Milne, Krish Menon, Mark McPhail, Marlies Ostermann, John A. Kellum, Gudrun Kunst

**Affiliations:** From the Department of Anaesthesia, King's College Hospital NHS Foundation Trust, London, UK (BM, GK), Institute of Liver Studies, King's College Hospital NHS Foundation Trust, London, UK (KM, MM), Department of Critical Care, Guy's & St Thomas’ NHS Foundation Trust, London, UK (MO), Department of Critical Care, University of Pittsburgh, USA (JAK), School of Cardiovascular and Metabolic Medicine & Sciences, King's College London, UK (GK)

Editor,

Acute kidney injury (AKI) is a common early complication of orthotopic liver transplantation (OLT), affecting up to 52% of patients within 72 h, associated with increased adverse outcomes.^1,2^ Identification of AKI is therefore important, but is predominantly dependent upon functional markers (serum creatinine (sCr) and urine output (UO)), which have generic and specific shortcomings to their use. In advanced liver disease, there is altered creatinine metabolism, whilst post-OLT, sCr concentration will be affected by fluid overload and UO may be uncoupled from glomerular filtration rate.^3^

Alternative renal biomarkers may prevent delayed or missed diagnosis of post-OLT AKI and permit the early implementation of evidence-based interventions, such as a renal care bundle, which has been shown to reduce incidence of AKI in high-risk patients after cardiac surgery.^4^ Elevated urinary cell cycle arrest biomarkers insulin-like growth factor-binding protein 7 (IGFBP7) and tissue inhibitor metalloproteinase-2 (TIMP-2) (combined to give a single product value (significant elevation considered to be [TIMP-2]×[IGFBP7] (T2I7) ≥0.3 ng ml^−2^/1000) have been described to be diagnostic for subclinical AKI (a pathological state of tubular damage without glomerular function loss) and predictive for subsequent KDIGO-defined AKI.^5^

Given the presence of an efficacious biomarker-guided AKI intervention, the use of these biomarkers should be further investigated in OLT patients. We conducted a single-centre observational study, measuring T2I7 preoperatively and at 6 h, 12 h, 24 h and 48 h after graft reperfusion. Our primary outcome was the difference in median urinary T2I7 biomarker levels pre- or postoperatively between patients that did and did not develop moderate (Stage 2) and severe (Stage 3) KDIGO-defined AKI within 72 h post-OLT.

We studied 20 adult patients (all of whom provided written informed consent) recruited to the observational iMET study between May and December 2021. Ethical approval for this study (19/NW/0750) was granted by the NHS North West – Haydock Research Ethics Committee (Manchester, UK) on 16 January 2020. Adult patients undergoing first-time single-organ OLT were eligible. Exclusion criteria included: significant pre-existing renal disease (eGFR < 70 ml min^−1^ 1.72 m^−2^) or AKI (by KDIGO Criteria within 1-month pre-operatively), significant cardiac disease (left ventricular ejection fraction <50%), patients undergoing ‘super-urgent’ OLT, and those receiving Level 2 or 3 organ support pre-operatively.

Pre-operative urine samples were analysed for T2I7 level (using an automated immunofluorescence assay (NephroCheck Test, BioMerieux SA, France & Astute140 Meter, Astute Medical Inc., USA), as were those at 6 h following intra-operative graft reperfusion (corresponding to within 2 h of arrival to critical care postoperatively), then at 12 h, 24 h and 48 h postreperfusion. Treating clinicians and participants were blinded to the biomarker values. Median biomarker values were compared between those patients developing/not developing KDIGO-defined AKI within 72 h using the Mann–Whitney *U* test. Logistic regression models and empirical receiver operating characteristic (ROC) curves were constructed for the prediction of subsequent AKI by biomarker value at each timepoint.

The incidence of all-stage KDIGO-defined AKI within 72 h of OLT was 80% (*n* = 16) with 55% developing moderate or severe AKI (*n* = 11). Baseline patient characteristics and operative factors were generally similar between those patients with and without moderate/severe AKI (Table 1, Table S1–S3, Supplemental Digital Content).

**Table 1:** Baseline characteristics and renal markers in patients developing or not developing moderate/severe acute kidney injury

	No moderate or severe AKI (45%; *n* = 9)	Moderate or severe AKI (55%; *n* = 11)	*P*-value
*Baseline patient characteristics*			
Age (years) median [IQR]	63 [58–67]	55 [48.5–64.5]	0.148
Male *n* (%)	6 (66.7%)	9 (81.8%)	0.795
Liver disease Aetiology *n* (%)			
ArLD	1 (11.1%)	6 (54.5%)	0.124
HCC	4 (44.4%)	1 (9.1%)	
AID	3 (33.3%)	2 (18.2%)	
NASH	1 (11.1%)	2 (18.2%)	
UKELD median [IQR]	49 [49–55]	55 [54.5–57.5]	0.079
*Baseline renal indices*			
Preoperative T2I7 [(ng ml^−1^)^2^/1000] Median [IQR]	0.32 [0.21–1.52]	1.27 [0.87–3.70]	0.047
Serum creatinine (μmol/l) mean (SD)	66.7 (8.7)	67.3 (16.3)	0.168
eGFR (ml/1.73 m^2^/min) median [IQR]	90 [75–90]	90 [86–90]	0.475
*Postoperative renal indices*			
ICU admission Serum creatinine (μmol/l) mean (SD)	68 (10.0)	76 (25.7)	0.359
KDIGO AKI (all-stage) by sCr criteria on ICU admission *n* (%)	0 (0)	1 (9.1%)	1.0
Post-reperfusion T2I7 [(ng/ml)^2^/1000] Median [IQR]			
6 h	0.30 [0.11–0.70]	1.58 [0.70–3.57]	0.025
12 h	0.42 [0.33–0.71]	1.24 [0.48–4.54]	0.047
24 h	0.19 [0.11–0.36]	0.89 [0.82–1.58]	0.002
48 h	0.12 [0.05–0.16]	0.76 [0.49–1.02]	0.001

AID, autoimmune disease; AKI, acute kidney injury; ArLD, alcohol-related liver disease; eGFR, estimated glomerular filtration rate; HCC, hepatocellular carcinoma; IQR, interquartile range; KDIGO, Kidney Disease Improving Global Outcomes; NASH, non-alcoholic steatohepatitis; sCr, serum creatinine; UKELD, United Kingdom Model for End-Stage Liver Disease.

Median T2I7 values were significantly higher at all perioperative timepoints in patients developing moderate to severe (Stage 2/3) AKI within 72 h (Table 1 and Fig. 1). In comparison, serum creatinine values were similar pre-operatively and on ICU admission between the two groups (Table 1). For patients developing all-stage KDIGO-defined AKI within 72 h, T2I7 values were significantly greater at 6 h, 12 h and 24 h postreperfusion (Table S4, Supplemental Digital Content and Figure S1, Supplemental Digital Content). Exploratory ROC curve analysis showed that the best balance of expedited diagnosis with performance characteristics for all-stage AKI was at 6 h (AUC 0.96 [0.88 to 1.00] with high sensitivity (0.88), specificity (1.00) and accuracy (0.90)) (Table S5 and Figures S2 and S3, Supplemental Digital Content).

**Fig. 1:**
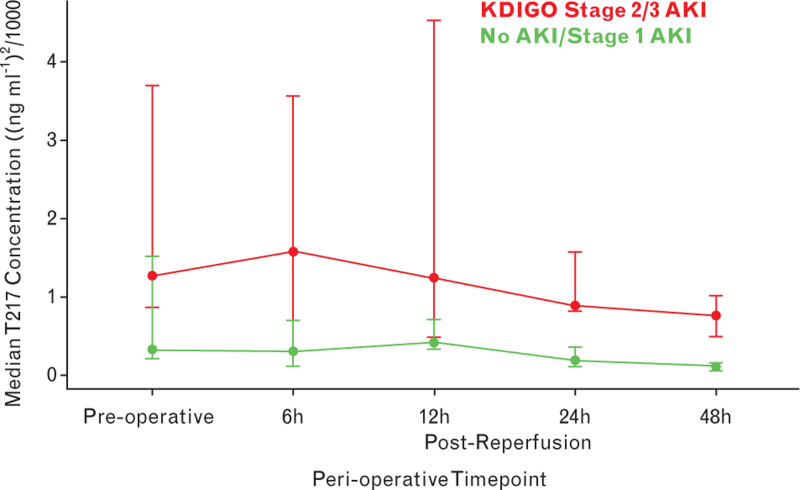
Median urinary T2I7 values in patients developing and not developing moderate to severe (Stage 2 or 3) AKI.

At each individual peri-operative timepoint, and taken as a whole, the biomarker concentrations were higher for patients subsequently developing moderate to severe AKI compared with those that did not. In AKI patients, the peak biomarker value was seen at 6 h postreperfusion. AKI, acute kidney injury.

Postoperatively, diagnosis of subclinical AKI (T2I7 ≥ 0.3 (ng ml^−1^)^2^/1000) occurred at a mean of 428 min postreperfusion (compared with 1048 min for KDIGO-defined AKI (*P* = 0.002)).

Our findings demonstrate that median urinary T2I7 values were significantly elevated pre-operatively and at all postreperfusion timepoints in patients developing moderate/severe post-OLT AKI. This suggests that they may identify renal risk, or subclinical disease, potentially pre-operatively, and may expedite diagnosis postoperatively. In contrast, serum creatinine was not significantly different between the two groups pre-operatively or on ICU admission. This highlights the possible potential of these biomarkers to implement early renal protective strategies.

These findings agree with the work of Fuhrman *et al.*, who in a paediatric cohort reported that urinary T2I7 was predictive of AKI post-OLT. Aside from the differences in age, this group similarly excluded patients with significant pre-existing renal disease.^6^ In contrast, Schiefer *et al.* reported that urinary T2I7 value was not predictive for post-OLT AKI, measuring levels at the end of surgery, and 24 h and 48 h postreperfusion.^7^ However, in our study we included earlier sampling timepoints (6 h and 12 h postreperfusion), which Fig. 1 (and Figure S1) suggest may be useful in discriminating patient trajectory. The rationale for earlier measurement was that these biomarkers are not dependent on gene transcription and are released early after, particularly, ischaemia-reperfusion injury, and may also reflect the sequelae of any intra-operative hypoperfusion or renal venous congestion.^8^ Furthermore, measurement at 6 h postreperfusion, in our study, corresponds to 2 h after ICU admission, providing a similar early risk stratification and potential for implementation of evidence-based renal protective strategies as in the PrevAKI-Multicenter study.^4^

Our study is primarily limited by its size, the high incidence of AKI and the relative homogeneity of its participants. However, our study included crucial early timepoints, and has given an indication as to the natural history of these biomarkers in this specific population, in patients without significant pre-existing renal disease exposed to differing surgical techniques and donor sources. In this study, even in the no moderate/severe AKI group, the T2I7 value was at points above the elevated threshold used in other studies, suggesting this patient cohort requires further examination.

In summary, median T2I7 values were higher at all peri-operative timepoints in patients developing AKI post-OLT, compared with those that did not. We suggest that these findings should prompt further studies, in a wider cohort of patients, focusing upon the 6 h postreperfusion timepoint. If confirmed, potential opportunities for early interventions to prevent moderate or severe AKI exist.

## Supplementary Material

Supplemental Digital Content
