# Supplementary material for: Cell cycle arrest biomarkers for early diagnosis of acute kidney injury after liver transplantation: A prospective cohort study
Source: Eur J Anaesthesiol. 2025 Jan 22;42(5):478–80. doi: 10.1097/EJA.0000000000002123 (PMC11970599; doi:10.1097/EJA.0000000000002123)

**Cell cycle arrest biomarkers for early diagnosis of acute kidney injury after liver transplantation**

Supplementary Material

Table S1. Baseline patient characteristics

|  | | **No AKI**  (n=4; 20%) | **AKI**  (n=16; 80%) | **P-value** |
| --- | --- | --- | --- | --- |
| Age *yr* Median [IQR] | | 64.5 [61.5 – 66.3] | 57 [48.8 – 65.5] | 0.3442 |
| Male % (n) | | 50% (2) | 81.3% (13) | 0.5186 |
| Ethnicity | White % (n) | 100% (4) | 81.3% (13) | 0.8756 |
|  | Asian % (n) | 0 (0) | 12.5% (2) | 1.0 |
|  | Mixed % (n) | 0 (0) | 6.3% (1) | 1.0 |
| ASA-PS % (n) | 2 | 0 (0) | 18.8% (3) | 0.5766 |
|  | 3 | 75% (3) | 68.8% (11) |  |
|  | 4 | 25% (1) | 12.5% (2) |  |
| Weight *kg* Mean (SD) | | 77.1 (13.9) | 84.6 (20.3) | 0.4127 |
| BMI *kg/m^2^* Median [IQR] | | 24.3 [22.6 – 28.6] | 26.5 [23.9 – 31.2] | 0.6167 |
| Cardiovascular System | Systolic BP *mmHg* Mean (SD) | 114 (13.9) | 121 (12.8) | 0.4408 |
|  | Peak VO_2_ *ml/kg/min* Median [IQR] | 16.9 [15.1 – 17.2] | 18.4 [15.8 – 23.7] | 0.1151 |
|  | AT *ml/kg/min* Median [IQR] | 11.8 [9.5 – 13.1] | 10.1 [8.8 – 12.4] | 1.0 |
|  | PCI % (n) | 0% (0) | 6.3% (1) | 1.0 |
| Liver Disease Aetiology % (n) | ArLD | 0% (0) | 43.8% (7) | 0.2915 |
|  | HCC | 50% (2) | 18.8% (3) | 0.5186 |
|  | Autoimmune | 50% (2) | 18.8% (3) | 0.5186 |
|  | NASH | 0% (0) | 18.8% (3) | 0.8756 |
| UKELD Median [IQR] | | 51 [47.5 – 54.3] | 55 [52 – 57.3] | 0.08693 |
| Comorbidities % (n) | CVS | 0% (0) | 37.5% (6) | 0.3932 |
|  | Respiratory | 50% (2) | 37.5% (6) | 1.0 |
|  | Neurological | 0% (0) | 6.3% (1) | 1.0 |
|  | GI | 0% (0) | 37.5% (6) | 0.3932 |
|  | Renal | 0% (0) | 12.5% (2) | 1.0 |
|  | Endocrine | 50% (1) | 43.8% (7) | 1.0 |
|  | Haem/Immune | 25% (1) | 56.3% (9) | 0.5762 |
|  | Other | 75% (3) | 12.5% (2) | 0.05281 |
| Nephrotoxic Drugs % (n) | Aminoglycosides | 0% (0) | 0% (0) | 1.0 |
|  | Diuretics | 50% (2) | 68.8% (11) | 0.9067 |
|  | ACEi | 0% (0) | 0% (0) | 1.0 |
|  | Other | 0% (0) | 6.3% (1) | 1.0 |
| Laboratory Values  Mean (SD)/Median [IQR] | Hb *g/L* | 125 (27.0) | 113 (22.4) | 0.4572 |
|  | Creatinine *umol/l* | 70 (6.3) | 66 (14.4) | 0.4134 |
|  | eGFR *ml/1.73m^2^/min* | 81.5 [74.5 – 88.5] | 90 [88.0 – 90.0] | 0.1075 |
|  | Albumin *g/l* | 41 [40 – 43] | 36 [30 – 39] | 0.05211 |
|  | Bilirubin *umol/l* | 23 [16 – 66] | 48 [33 – 81] | 0.3947 |
|  | AST *U/l* | 51 [32 – 98] | 56 [46 – 95] | 0.7766 |
|  | ALP *U/l* | 110 [94 – 276] | 197 [100 – 237] | 0.7505 |
|  | GGT *U/l* | 81 [63 – 112] | 65 [33 – 145] | 0.5702 |

Table S2. Surgical Factors, Graft Characteristics and Intraoperative Management

|  |  | **No AKI**  (n=4; 20%) | **AKI**  (n=16; 80%) | **P-value** |
| --- | --- | --- | --- | --- |
| Surgical Technique % (n) | Caval Replacement | 75% (3) | 50% (8) | 0.736 |
|  | Piggyback | 25% (1) | 50% (8) |  |
| Anastomosis % (n) | Duct-Duct | 75% (3) | 87.5% (14) | 1.0 |
|  | Hep-Jej | 25% (1) | 12.5% (2) |  |
| Donor % (n) | DBD | 50% (2) | 75% (12) | 0.7144 |
|  | DCD | 50% (2) | 25% (4) |  |
| Graft | Weight *g* Mean (SD) | 1510 (321) | 1772 (354) | 0.2136 |
|  | No Steatosis % (n) | 100% (4) | 62.5% (10) | 0.3932 |
|  | Mild Steatosis % (n) | 0% (0) | 18.8% (3) | 0.8756 |
|  | Moderate Steatosis % (n) | 0% (0) | 18.8% (3) | 0.8756 |
|  | Cold Ischaemic Time *min* Mean (SD) | 439 (106) | 489 (56) | 0.5057 |
| Intraoperative | Duration *min* Mean (SD) | 381 (40) | 369 (73) | 0.6724 |
|  | Peak MAP *mmHg* Mean (SD) | 116 (23) | 114 (17) | 0.8778 |
|  | Nadir MAP *mmHg* Mean (SD) | 54 (2.5) | 48 (7) | 0.0407 |
|  | Noradrenaline dose *mg* Median [IQR] | 2.06 [0.99 – 3.40] | 1.32 [0.94 – 2.52] | 0.5965 |
|  | Adrenaline dose *mg* Median [IQR] | 0.04 [0.0 – 0.08] | 0.02 [0.0 – 0.07] | 1.0 |
|  | Terlipressin dose *mg* Median [IQR] | 0.0 [0.0 – 0.3] | 0.0 [0.0 – 1.0] | 0.6797 |
|  | Crystalloid Vol *ml* Median [IQR] | 3750 [2750 – 4500] | 2000 [1500 – 4000] | 0.4942 |
|  | Colloid Vol *ml* Median [IQR] | 1250 [375 – 2125] | 500 [500 – 1250] | 0.8772 |
|  | Albumin Vol *ml* Median [IQR] | 0 [0 – 0] | 0 [0 – 0] | 0.5115 |
|  | ICS Blood Vol *ml* Median [IQR] | 257 [0.0 – 610] | 515 [247 – 835] | 0.34 |
|  | PRC Vol *ml* Median [IQR] | 285 [0.0 – 653] | 329 [0.0 – 1155] | 0.7161 |
|  | Platelet Vol *ml* Median [IQR] | 138 [0.0 – 283] | 231 [0.0 – 506] | 0.4994 |
|  | FFP Vol *ml* Median [IQR] | 1101 [821 – 1291] | 1105 [761 – 2207] | 0.6879 |
|  | Cryoprecipitate Vol *ml* Median [IQR] | 0.0 [0.0 – 0.0] | 0.0 [0.0 – 449] | 0.1188 |
|  | EBL *ml* Median [IQR] | 2750 [2000 – 3625] | 3900 [2000 – 5500] | 0.5317 |
|  | Total UO *ml/kg/h* Median [IQR] | 1.06 [0.92 – 1.60] | 0.75 [0.34 – 1.03] | 0.1841 |
|  | Lowest UO *ml/kg/h* Median [IQR] | 0.82 [0.53 – 1.16] | 0.54 [0.24 – 0.76] | 0.1839 |
|  | CBG *>10mmol/L* % (n) | 50% (2) | 56.3% (9) | 1.0 |

Table S3. Postoperative Management and Outcomes

|  | | **No AKI**  (n=4; 20%) | **AKI**  (n=16; 80%) | **P-value** |
| --- | --- | --- | --- | --- |
| ICU LOS *hr* Median [IQR] | | 43 [39.5 – 45] | 48 [43-67] | 0.2365 |
| MV Duration *hr* Median [IQR] | | 16 [14 – 17] | 15 [11 - 19] | 0.9241 |
| MAP *mmHg* Mean (SD) | Peak | 102 (11) | 113 (16) | 0.1636 |
|  | Nadir | 65 (6) | 63 (6) | 0.5851 |
| Noradrenaline Dose *mcg/kg/min* Median [IQR] | Day 0 | 0.01 [0.00 – 0.04] | 0.08 [0.05 – 0.10] | 0.1539 |
|  | Day 1 | 0.02 [0.00 – 0.005] | 0.00 [0.00 – 0.05] | 0.7897 |
|  | Day 2 | 0.00 [0.00 – 0.00] | 0.00 [0.00 – 0.00] | 0.7077 |
| Crystalloid Volume *ml* Mean (SD) | Day 0 | 2685 (1454) | 2257 (1279) | 0.617 |
|  | Day 1 | 1836 (385) | 2485 (902) | 0.04854 |
|  | Day 2 | 1200 (1239) | 981 (910) | 0.7577 |
|  | Day 3 | 1510 (1080) | 1057 (990) | 0.485 |
| Colloid Volume *ml* Mean (SD) | Day 0 | 0.0 [0.0 – 25.0] | 0.0 [0.0 – 0.0] | 0.8922 |
|  | Day 1 | 0.0 [0.0 – 0.0] | 0.0 [0.0 – 0.0] | 0.5256 |
|  | Day 2 | - | - | - |
|  | Day 3 | 0.0 [0.0 – 0.0] | 0.0 [0.0 – 0.0] | 0.7077 |
| Transfusion Volume *ml* Mean (SD) | PRC | 0 [0 – 0] | 152 [0 – 644] | 0.09815 |
|  | Platelet | 0 [0 – 0] | 0 [0 – 226] | 0.1803 |
|  | FFP | 0 [0 – 0] | 0 [0 – 0] | 0.5256 |
|  | Cryo | 0 [0 – 0] | 0 [0 – 0] | 0.4032 |
| Standard Immunosuppression % (n) | | 100% (4) | 75% (12) | 0.675 |
| Total UO *ml/kg/h* Mean (SD) | Day 1 | 1.05 (0.19) | 0.61 (0.35) | 0.00854 |
|  | Day 2 | 1.05 (1.16) | 0.94 (0.78) | 0.7163 |
|  | Day 3 | 1.42 (0.81) | 1.30 (1.03) | 0.8122 |
| Lowest UO *ml/kg/h* Mean (SD) | Day 1 | 0.26 [0.24 – 0.31] | 0.11 [0.05 – 0.24] | 0.09619 |
|  | Day 2 | 0.36 [0.27 – 0.37] | 0.25 [0.08 – 0.34] | 0.4182 |
|  | Day 3 | 0.42 [0.37 – 0.44] | 0.39 [0.19 – 0.64] | 1.0 |
| Peak Laboratory Values Mean (SD) | Bilirubin *umol/l* | 88 (52) | 77 (39) | 0.7276 |
|  | Creatinine *umol/l* | 79 (8) | 144 (53) | 0.0002 |
| Hospital LOS *days* Median [IQR] | | 14 [12 – 15] | 14 [12 – 15] | 1.0 |
| Mortality % (n) | In-Hospital | 0 | 0 | 1.0 |
|  | 30-day | 0 | 0 | 1.0 |

Table S4. Difference in median [TIMP-2]x[IGFBP7] levels for patients developing all-stage AKI vs no AKI

|  | | **No AKI**  (n=4; 20%) | **AKI**  (n=16; 80%) | **P-value** |
| --- | --- | --- | --- | --- |
| Preoperative *(ng/ml)^2^/1000*  Median [IQR] | | 0.55 [0.29 – 0.97] | 1.16 [0.74 – 2.48] | 0.2485 |
| Post-Reperfusion *(ng/ml)^2^/1000*  Median [IQR] | 6h | 0.10 [0.08 – 0.13] | 1.00 [0.50 – 2.15] | 0.00612 |
|  | 12h | 0.30 [0.23 – 0.45] | 0.64 [0.44 – 3.44] | 0.04995 |
|  | 24h | 0.12 [0.10 – 0.16] | 0.83 [0.39 – 1.27] | 0.01682 |
|  | 48h | 0.17 [0.10 – 0.22] | 0.37 [0.15 – 0.85] | 0.2215 |

Table S5. Comparison of predictive ability of [TIMP-2]x[IGFBP7] for all-stage AKI or severe AKI comparing area under the receiver operator characteristic curve, and sensitivity, specificity and accuracy, using the cut-off value of $\geq$0.3(ng/ml)^2^/1000 to identify a positive test

|  | | **ROC Curve** | **Performance** | | |
| --- | --- | --- | --- | --- | --- |
|  |  | AUC [95% CI] | Sensitivity | Specificity | Accuracy |
| **All Stage AKI** | Baseline | 0.70 [0.42 – 0.99] | 0.81 | 0.25 | 0.7 |
|  | 6h | 0.96 [0.88 – 1.0] | 0.88 | 1.00 | 0.9 |
|  | 12h | 0.83 [0.55 – 1.0] | 1.00 | 0.50 | 0.9 |
|  | 24h | 0.92 [0.79 – 1.0] | 0.88 | 1.00 | 0.9 |
|  | 48h | 0.75 [0.52 – 0.98] | 0.56 | 1.00 | 0.65 |
| **Severe (Stage 2/3) AKI** | Baseline | 0.77 [0.54 – 1.0] | 1.00 | 0.44 | 0.75 |
|  | 6h | 0.80 [0.60 – 1.0] | 0.82 | 0.44 | 0.65 |
|  | 12h | 0.77 [0.55 – 0.99] | 1.00 | 0.22 | 0.65 |
|  | 24h | 0.94 [0.84 – 1.0] | 1.00 | 0.67 | 0.85 |
|  | 48h | 0.98 [0.92 – 1.0] | 0.82 | 1.00 | 0.9 |

Figure S1. Median urinary [TIMP-2]x[IGFBP7] concentration in patients developing and not developing all-stage AKI. At each individual perioperative timepoint the urinary biomarker concentration is higher in patients that subsequently developed AKI. At 6h post-reperfusion there is particularly marked difference in the concentrations.


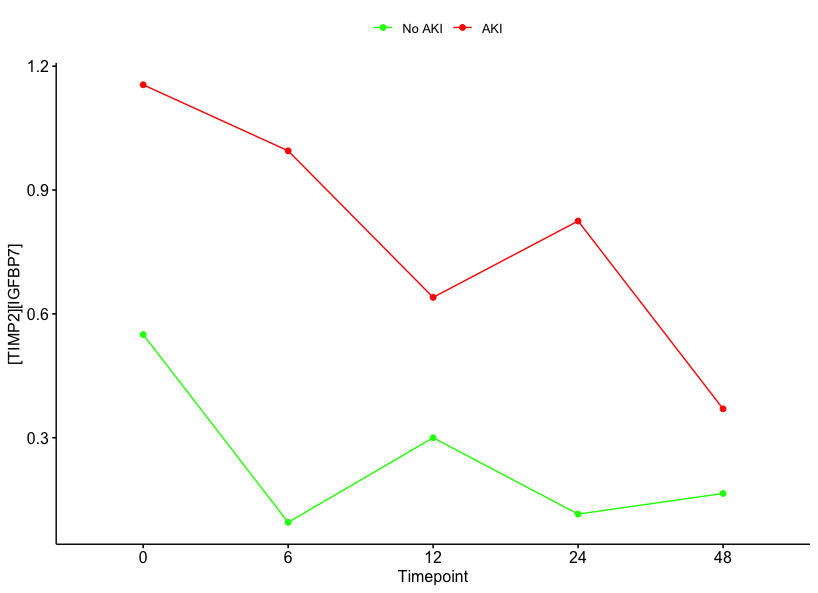


Figure S2. ROC curves for development of moderate to severe (stage 2/3) AKI by [TIMP-2]x[IGFBP7] measurement at different perioperative timepoints.


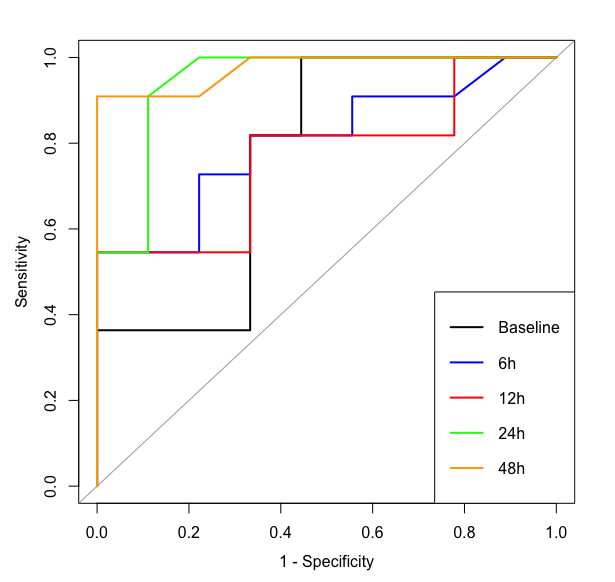


Figure S3. ROC curves for development of all-stage AKI by [TIMP-2]x[IGFBP7] values at different perioperative timepoints.


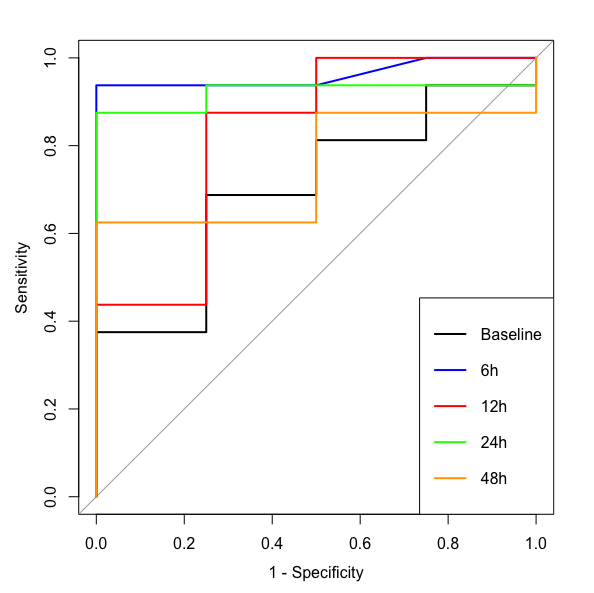

Supplement: Supplemental Digital Content [file ejanet-42-478-s001.docx]
